# Novel Chromosome-Borne Accessory Genetic Elements Carrying Multiple Antibiotic Resistance Genes in *Pseudomonas aeruginosa*

**DOI:** 10.3389/fcimb.2021.638087

**Published:** 2021-03-18

**Authors:** Ting Yu, Huiying Yang, Jun Li, Fangzhou Chen, Lingfei Hu, Ying Jing, Xinhua Luo, Zhe Yin, Mingxiang Zou, Dongsheng Zhou

**Affiliations:** ^1^Department of Clinical Laboratory, Xiangya Hospital, Central South University, Changsha, China; ^2^State Key Laboratory of Pathogen and Biosecurity, Beijing Institute of Microbiology and Epidemiology, Beijing, China

**Keywords:** *Pseudomonas aeruginosa*, *β*-lactam resistance, mobile genetic elements, *bla*_CARB-53_, *catB3s*, ST3405

## Abstract

*Pseudomonas aeruginosa* is noted for its intrinsic antibiotic resistance and capacity of acquiring additional resistance genes. In this study, the genomes of nine clinical *P. aeruginosa* isolates were fully sequenced. An extensive genetic comparison was applied to 18 *P. aeruginosa* accessory genetic elements (AGEs; 13 of them were sequenced in this study and located within *P. aeruginosa* chromosomes) that were divided into four groups: five related integrative and conjugative elements (ICEs), four related integrative and mobilizable elements (IMEs), five related unit transposons, and two related IMEs and their two derivatives. At least 45 resistance genes, involved in resistance to 10 different categories of antibiotics and heavy metals, were identified from these 18 AGEs. A total of 10 *β*-lactamase genes were identified from 10 AGEs sequenced herein, and nine of them were captured within class 1 integrons, which were further integrated into ICEs and IMEs with intercellular mobility, and also unit transposons with intracellular mobility. Through this study, we identified for the first time 20 novel MGEs, including four ICEs Tn*6584*, Tn*6585*, Tn*6586*, and Tn*6587*; three IMEs Tn*6853*, Tn*6854*, and Tn*6878*; five unit transposons Tn*6846*, Tn*6847*, Tn*6848*, Tn*6849*, and Tn*6883*; and eight integrons In1795, In1778, In1820, In1784, In1775, In1774, In1789, and In1799. This was also the first report of two resistance gene variants *bla*_CARB-53_ and *catB3s*, and a novel ST3405 isolate of *P. aeruginosa*. The data presented here denoted that complex transposition and homologous recombination promoted the assembly and integration of AGEs with mosaic structures into *P. aeruginosa* chromosomes.

## Introduction

*Pseudomonas aeruginosa* is a major opportunistic pathogen and a leading cause of morbidity and mortality in cystic fibrosis patients and immunocompromised individuals (Alvarez-Ortega et al., 2011). *P. aeruginosa* displays resistance to multiple classes of antibiotics (De Oliveira et al., 2020), being in the critical priority list of antibiotic resistant bacteria created by the World Health Organization (Tacconelli et al., 2018). One of the main mechanisms driving to the antibiotic resistance in *P. aeruginosa* is the production of *β*-lactamases, which are capable of hydrolyzing chemical compounds containing a *β*-lactam ring (Bush, 2018) and are the most common determinant for resistance to bacterial *β*-lactam antibiotics (Bush and Bradford, 2020). *β*-lactamases can be divided into four Ambler classes A to D, where classes A/C/D *β*-lactamases utilize a serine moiety while class B *β*-lactamases (also known as metallo-*β*-lactamases) need a zinc ion at its active site (Ambler, 1980). Besides the intrinsic narrow-spectrum *β*-lactamase (NSBL) gene *bla*_OXA-50_ (Girlich et al., 2004), *P. aeruginosa* has evolved to acquire various extended-spectrum *β*-lactamase (ESBL) and even carbapenemase genes through horizontal gene transfer mediated by different mobile genetic elements (MGEs) (Botelho et al., 2019). MGEs play critical roles in the accumulation and spread of antibiotic resistance genes in *P. aeruginosa* (Botelho et al., 2019).

Among these genetic platforms, the integrative and conjugative elements (ICEs) are transposons which encode self-integration and -conjugation modules, typically including *attL* (attachment site at the left end), *int* (integrase), *xis* (excisionase), *rlx* (relaxase), *oriT* (origin of conjugative replication), *cpl* (coupling protein), a P (TivB)- or F (TivF)-type T4SS gene set (mating pair formation), and *attR* (attachment site at the right end) (Botelho and Schulenburg, 2020). Conversely, the integrative and mobilizable elements (IMEs) (that typically have *attL*, *int*, *rlx*, *oriT*, and *attR*) do not encode the T4SS machinery, and their mobility needs a helper conjugative element (Guédon et al., 2017). The Tn*3*-family unit transposons carry a core transposition module typically composed of *tnpA* (transposase), *tnpR* (resolvase), and *res* (resolution site), with a pair of terminal inverted repeats IRL/IRR (Nicolas et al., 2015).

Various ICEs, IMEs, and unit transposons have been identified and found to be important vehicles for antibiotic resistance genes in *P. aeruginosa* (Stokes et al., 2007; Mathee et al., 2008; Roy Chowdhury et al., 2016; Botelho et al., 2018a; Botelho et al., 2018b; van der Zee et al., 2018; Chew et al., 2019). Deep understanding of the MGEs at a genomic level will provide the theoretical basis for inhibiting the emergence of multidrug-resistant *P. aeruginosa* and overcoming the challenge of limited antimicrobial chemotherapy measures. However, few studies have dedicated to detailedly and accurately dissect the genetic structures of these MGEs from *P. aeruginosa*.

Our previous studies (Zhan et al., 2018; Zeng et al., 2019) have dissected the genetic characteristics of three carbapenemase-encoding novel transposons (*bla*_IMP-1_-containing IS*Pa17*-based transposition unit Tn*6394*, *bla*_VIM-4_-containing ICE Tn*6413*, and *bla*_IMP-1_-containing unit transposon Tn*6411*) located within *P. aeruginosa* chromosomes. This follow-up study presented the complete sequences of 10 *β*-lactamase-encoding accessory genetic elements (AGEs; encoding four NSBLs CARB-2/-53, TEM-1B, and OXA-101; four ESBLs GES-1, PER-1, VEB-3 and OXA-10; and two carbapenemases GES-6/-15) along with three additional ones that did not encode any *β*-lactamase genes (but harbored *dfrA12* and *aphA6* and *strAB*) from the chromosomes of the sequenced *P. aeruginosa* strains. A detailed genetic dissection was applied to these 13 AGEs together with additional five reference/prototype ones from GenBank. Data presented here provided a deeper understanding of diversification of chromosomal MGEs (especially those carrying *β*-lactamase genes) in *P. aeruginosa*.

## Materials and Methods

### Bacterial Strains

Nine *P. aeruginosa* isolates (**Table S1**) were recovered either from sputum, airway secretions, urine, or blood of nine patients with nosocomial infections in five different Chinese public hospitals from 2011 to 2019. Bacterial species identification was performed using genome sequence-based average nucleotide identity analysis (http://www.ezbiocloud.net/tools/ani) (Richter and Rossello-Mora, 2009).

### Sequencing and Sequence Assembly

Bacterial genomic DNA was isolated using the UltraClean Microbial Kit (Qiagen, NW, Germany) and sequenced from a sheared DNA library with average size of 15 kb (ranged from 10 to 20 kb) on a PacBio RSII sequencer (Pacific Biosciences, CA, USA), as well as a paired-end library with an average insert size of 350 bp (ranged from 150 to 600 bp) on a HiSeq sequencer (Illumina, CA, USA). The paired-end short Illumina reads were used to correct the long PacBio reads utilizing *proovread* (Hackl et al., 2014), and then the corrected PacBio reads were assembled *de novo* utilizing *SMARTdenovo* (https://github.com/ruanjue/smartdenovo).

### Sequence Annotation and Comparison

Open reading frames (ORFs) and pseudogenes were predicted using *RAST* 2.0 (Brettin et al., 2015) combined with *BLASTP/BLASTN* searches (Boratyn et al., 2013) against the *UniProtKB/Swiss-Prot* database (Boutet et al., 2016) and the *RefSeq* database (O’Leary et al., 2016). Annotation of resistance genes, MGEs, and other features was carried out using the online databases including *CARD* (Jia et al., 2017), *ResFinder* (Zankari et al., 2012), *ISfinder* (Siguier et al., 2006), *INTEGRALL* (Moura et al., 2009), and *Tn Number Registry* (Roberts et al., 2008). Multiple and pairwise sequence comparisons were performed using *MUSCLE* 3.8.31 (Edgar, 2004) and *BLASTN*, respectively. Gene organization diagrams were drawn in *Inkscape* 1.0 (https://inkscape.org/en/). Heatmaps were plotted with *MeV* 4.9.0 (Saeed et al., 2003).

### Multi-Locus Sequence Typing

The sequence types (STs) of *P. aeruginosa* isolates were identified according to the online *P. aeruginosa* MLST scheme (https://pubmlst.org/paeruginosa/).

### Conjugal Transfer

Conjugal transfer experiments were carried out with rifampin-resistant *P. aeruginosa* PAO1 being used as a recipient, and the indicated wild-type *P. aeruginosa* isolate as a donor. Three milliliters of overnight cultures of each of donor and recipient bacteria was mixed together, harvested, and resuspended in 80 μL of Brain Heart Infusion (BHI) broth (BD Biosciences). The mixture was spotted on a 1 cm^2^ hydrophilic nylon membrane filter with a 0.45 µm pore size (Millipore) that was placed on BHI agar (BD Biosciences) plate and then incubated for mating at 37°C for 12 to 18 h. Bacteria were washed from filter membrane and spotted on Muller–Hinton (MH) agar (BD Biosciences) plates, for selecting a *bla*_GES_- or *bla*_CARB_-carrying PAO1 transconjugant. 1500 mg/mL rifampin (for PAO1), together with 80 mg/mL ceftazidime (for *bla*_GES_) or 200 mg/L carbenicillin (for *bla*_CARB_) was used for transconjugant selection.

### Cloning Experiments

The *bla*_CARB-53_ coding region together with its 365-bp upstream (promoter) region and 305-bp downstream (terminator) region from strain 201330 was cloned into the cloning vector pUC57-Kan (pUC57K). Similarly, the *catB3s* coding region together with its 309-bp upstream (promoter) region and 260-bp downstream (terminator) region from strain YT12746 was cloned into pUC57K. The resulting recombinant plasmid pUC57K-CARB or pUC57K-catB was transformed through electroporation into *Escherichia coli* TOP10, generating the *E. coli* electroporant TOP10/pUC57K-CARB or TOP10/pUC57K-catB, respectively. 200 μg/mL ampicillin (for *bla*_CARB-53_) or 25 μg/mL chloramphenicol (for *catB3s*) was used for electroporant selection.

### Bacterial Antimicrobial Susceptibility Test

Bacterial antimicrobial susceptibility was tested by VITEK 2, E-test, or the classic broth microdilution method and interpreted as per the 2020 Clinical and Laboratory Standards Institute (CLSI) guidelines (CLSI, 2020).

### Nucleotide Sequence Accession Numbers

The complete chromosome sequences of the SE5352, SE5331, 31130, 201330, T12726, YTSEY8, SE5429, SE5458, and SE5357 isolates were submitted to GenBank under accession numbers CP054843, CP046402, CP060392, CP054623, CP045552, CP054581, CP054845, CP046406, and CP054844 respectively.

## Results and Discussion

### Identification of Four STs From the Nine Clinical *P. aeruginosa* Isolates

Four different STs, namely ST235, ST244, ST292, and ST3405, were identified from the nine *P. aeruginosa* isolates analyzed in this work (**Table S1**). The five isolates SE5352, SE5331, 31130, SE5429, and SE5458, and the SE5357 isolate belonged to ST235 and ST244, respectively, which were recognized as the high-risk clones with ST235 being the most widely disseminated (Oliver et al., 2015). The YT12746 isolate belonged to ST292, a *P. aeruginosa* clone type identified in Asian countries only such as Korea (Lee et al., 2011), China (Fan et al., 2016), and Thailand (Khuntayaporn et al., 2019). The 201330 isolate belonged to a novel ST variant ST3405, with an allelic profile 17-5-5-4-137-4-3 corresponding to the seven housekeeping genes *acsA*, *aroE*, *guaA*, *mutL*, *nuoD*, *ppsA*, and *trpE*.

### Identification of 13 Chromosomal AGEs From the Nine Clinical *P. aeruginosa* Isolates

Based on the determined complete genome sequences of the nine *P. aeruginosa* isolates, a total of 13 chromosomal AGEs were identified for further genetic characterization: *bla*_GES-1/-6/-15_-carrying Tn*6584*, Tn*6585*, and Tn*6586* from SE5352, SE5331, and 31130, respectively; *bla*_CARB-2/-53_-carrying Tn*6847* and Tn*6587* from YTSEY8 and 201330, respectively; *bla*_VEB-3_-carrying Tn*6878* and ‘*bla*_VEB-3_ region’ from SE5429 and SE5458, respectively; *bla*_PER-1_-carrying Tn*6846* and Tn*6853* (containing Tn*6848*) from SE5357 and YT12746, respectively; *dfrA12*- and *aphA6*-carrying ‘*dfrA12* region’ from SE5458; and *strAB*-carrying Tn*6854* (containing Tn*6849*) from YTSEY8 (**Table 1**).

**Table 1:** Major features of AGEs characterized in this work.

Group	Mobile element	Accession number	*β*-lactamase gene	Ambler class	Location (nucleotide position)	Length(bp)	Host bacterium	Ref.
Tn*6417*- related ICEs	Tn*6417*	CP013993	None		Chromosome (5365108.5473293)	108,186	*P. aeruginosa* DHS01	(Valot et al., 2014)
	Tn*6584*	CP054843	*bla*_GES-1_	A	Chromosome (5210218.5310913)	100,696	*P. aeruginosa* SE5352	This study
	Tn*6585*	CP046402	*bla*_GES-6_	A	Chromosome (5360752.5461449)	100,698	*P. aeruginosa* SE5331	This study
	Tn*6586*	CP060392	*bla*_GES-15_	A	Chromosome (5210194.5310891)	100,698	*P. aeruginosa* 31130	This study
	Tn*6587*	CP054623	*bla*_CARB-53_	A	Chromosome (4283536.4404385)	120,850	*P. aeruginosa* 201330	This study
Tn*6852*- related IMEs	Tn*6852*	HG530068	None		Chromosome (6925701.6939682)	13,982	*P. aeruginosa* PA38182	(Witney et al., 2014)
	Tn*6853*	CP045552	*bla*_PER-1_ *bla*_CARB-2_	A	Chromosome (1159847.1238980)	79,134	*P. aeruginosa* YT12746	This study
	Tn*6854*	CP054581	None		Chromosome (1080738.1148847)	68,110	*P. aeruginosa* YTSEY8	This study
	Tn*6855*	MK347425	None		Plasmid pHNAH8I-1 (61489.77177)	15,689	*K. pneumoniae* AHM7C8I	(Lv et al., 2020)
Tn*1403*- related unit transposons	Tn*1403*	AF313472	*bla*_CARB-2_	A	Plasmid RPL11 (Not applicable)	19,630	*P. aeruginosa * RPL11	(Stokes et al., 2007)
	Tn*6846*	CP054844	*bla*_PER-1_	A	Chromosome (6080493.6110780)	30,288	*P. aeruginosa * SE5357	This study
			*bla*_OXA-101_	D				
	Tn*6847*	CP054581	*bla*_CARB-2_	A	Chromosome (3311175.3339962)	28,788	*P. aeruginosa * YTSEY8	This study
	Tn*6848*	CP045552	*bla*_PER-1_ *bla*_CARB-2_	A	Chromosome (1165906.1229345)	63,440	*P. aeruginosa * YT12746	This study
	Tn*6849*	CP054581	None		Chromosome (1086797.1139212)	52,416	*P. aeruginosa * YTSEY8	This study
Tn*6877*- related IMEs	Tn*6877*	CP021775	*bla*_GES-1_	A	Chromosome (4994559.5050439)	55,881	*P. aeruginosa* Pa58	(Espinosa-Camacho et al., 2017)
			*bla*_OXA-2_	D				
	Tn*6878*	CP054845	*bla*_VEB-3_	A	Chromosome (4863462.4926615)	63,154	*P. aeruginosa* SE5429	This study
			*bla*_OXA-10_	D				
	*dfrA12* region	CP046406	None		Chromosome (2150652.2198200)	47,549	*P. aeruginosa* SE5458	This study
	*bla*_VEB-3_ region	CP046406	*bla*_VEB-3_ *bla*_TEM-1B_	A	Chromosome (4660362.4688297)	27,936	*P. aeruginosa* SE5458	This study
			*bla*_OXA-10_	D				

Listed were the 13 chromosomal AGEs from the nine P. aeruginosa isolates sequenced in this study, together with the five reference MGEs from the GenBank. Notably, Tn6848, Tn6849, Tn6882, and Tn6883 were inserted within Tn6853, Tn6854, Tn6877, and Tn6878, respectively. Two chromosome-borne MGEs—the previously characterized mer-carrying unit transposon Tn6488 (Jiang et al., 2020) found in the isolates YT12746 and YTSEY8, and the IME Tn6779 (carrying no resistance genes; unrelated to this study) from the SE5357 isolate—were excluded.

### Collection of 18 AGEs for Sequence Comparison

**Figure 1:**
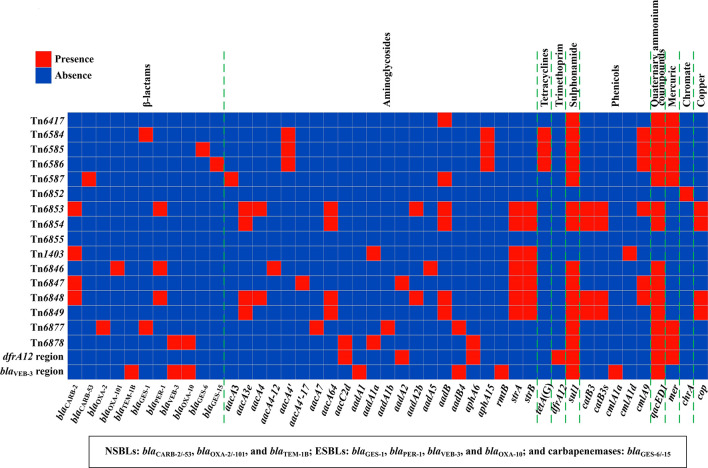
Heatmap of prevalence of resistance genes. The original data are shown in **Table S2**.

A detailed sequence comparison was then applied to a collection of four groups of 18 AGEs including the above 13 chromosomal AGEs together with five additional reference/prototype ones Tn*6417*, Tn*6852*, Tn*6855*, Tn*6877*, and Tn*1403* from the GenBank: five related ICEs Tn*6417*, Tn*6584*, Tn*6585*, Tn*6586*, and Tn*6587*; four related IMEs Tn*6852*, Tn*6853*, Tn*6854*, and Tn*6855*; five related unit transposons Tn*1403*, Tn*6846*, Tn*6847*, Tn*6848*, and Tn*6849*; and two related IMEs Tn*6877* and Tn*6878* and their two derivatives *dfrA12* region and *bla*_VEB-3_ region (**Table 1**). At least 45 resistance genes, involved in resistance to 10 different categories of antibiotics and heavy metals, were identified in 17 of these 18 AGEs (**Figure 1** and **Table S2**).

### Comparison of Five Related ICEs Tn*6417*, Tn*6584*, Tn*6585*, Tn*6586*, and Tn*6587*

**Figure 2:**
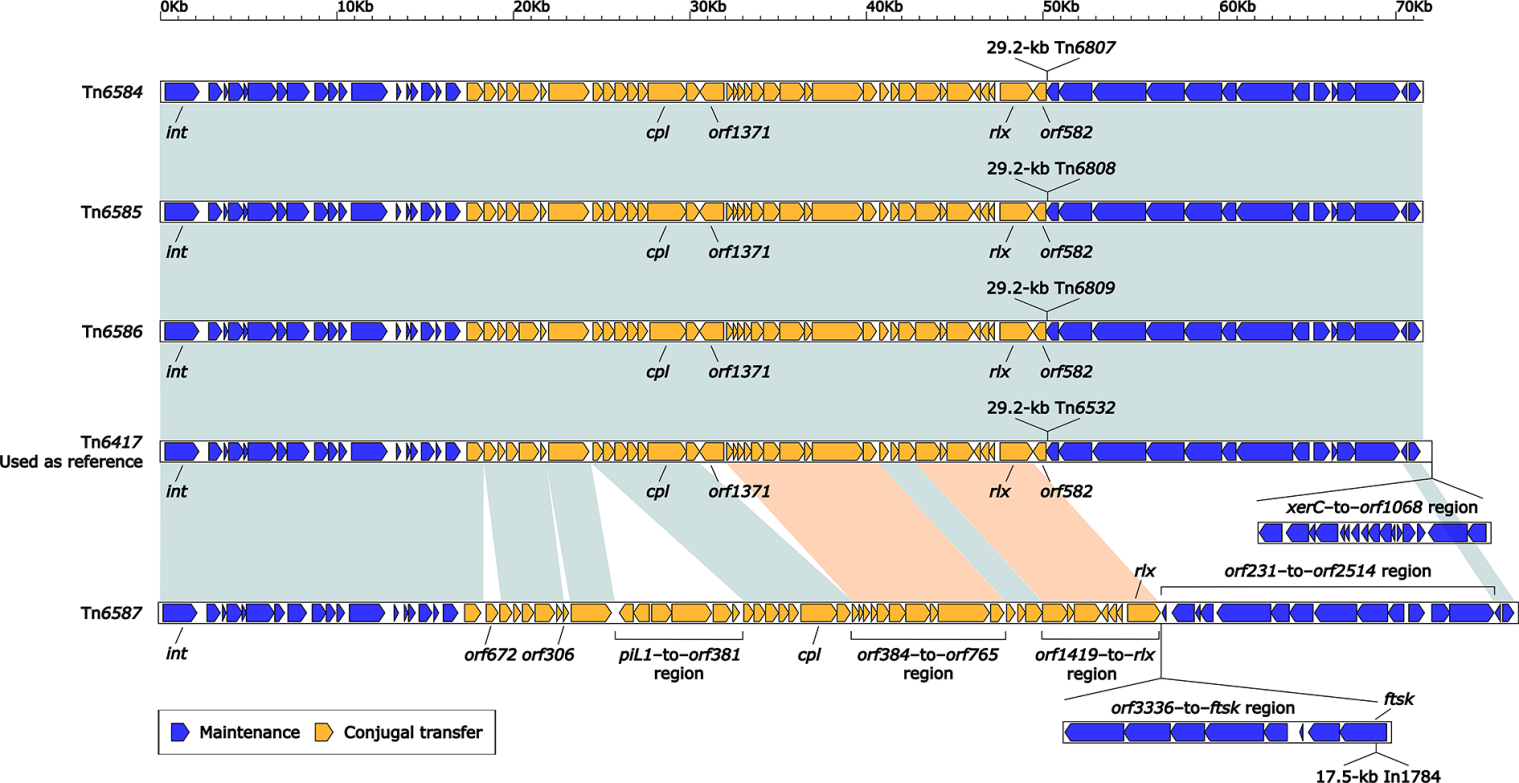
Comparison of five related ICEs Tn*6417*, Tn*6584*, Tn*6585*, Tn*6586*, and Tn*6587*. Genes are denoted by arrows. Genes, MGEs, and other features are colored based on their functional classification. Shading in light blue denotes regions of homology (nucleotide identity ≥90%), light orange (nucleotide identity <90%). The accession number of Tn*6417* (Valot et al., 2014) used as reference is EU696790.

Tn*6417* (108.2 kb in length) was used as reference ICE (Zeng et al., 2019) and initially described in *P. aeruginosa* DHS01 (Valot et al., 2014). The backbones of Tn*6417*, Tn*6584*, Tn*6585*, Tn*6586*, and Tn*6587* varied in size from about 71.5 kb to nearly 103.3 kb, but all contained *attL/R*, *int*, *cpl*, *rlx*, and a F-type T4SS gene set (**Figure 2**). In addition, their backbones had at least four major modular differences: i) presence of unique *orf672*, *orf306*, *piL1*–to–*orf381* region, *orf3336*–to–*ftsk* region, and *orf231*–to–*orf2514* region in only Tn*6587*; ii) presence of unique *xerC*–to–*orf1068* region in only Tn*6417*; iii) absence of *orf1371* and *orf582* from only Tn*6587*; and iv) *orf384*–to–*orf765* region and *orf1419–*to*–rlx* region from Tn*6587* displayed <90% nucleotide identity to the counterparts of Tn*6417*, Tn*6584*, Tn*6585*, and Tn*6586* (**Figure 2**). Tn*6417*, Tn*6584*, Tn*6585*, and Tn*6586* were integrated into the chromosomal tRNA^Gly^ gene, while Tn*6587* into the chromosomal gene *tolC* (outer membrane protein). The *attL*/*R* sequences of these five ICEs somewhat showed differences but shared a core motif ‘TTCGCCCGCTCCA’.

Each of these five ICEs carried a single accessory module: Tn*6807*, Tn*6808*, Tn*6809*, Tn*6532*, and In*1784* in Tn*6584*, Tn*6585*, Tn*6586*, Tn*6417*, and Tn*6587*, respectively (**Figure 2**). The former four were integrated at a site upstream of the ICE backbone gene *orf582* and identified as derivatives of Tn*6346* (Ng et al., 2009), while the last one into the ICE backbone gene *ftsk* (cell division protein).

**Figure 3:**
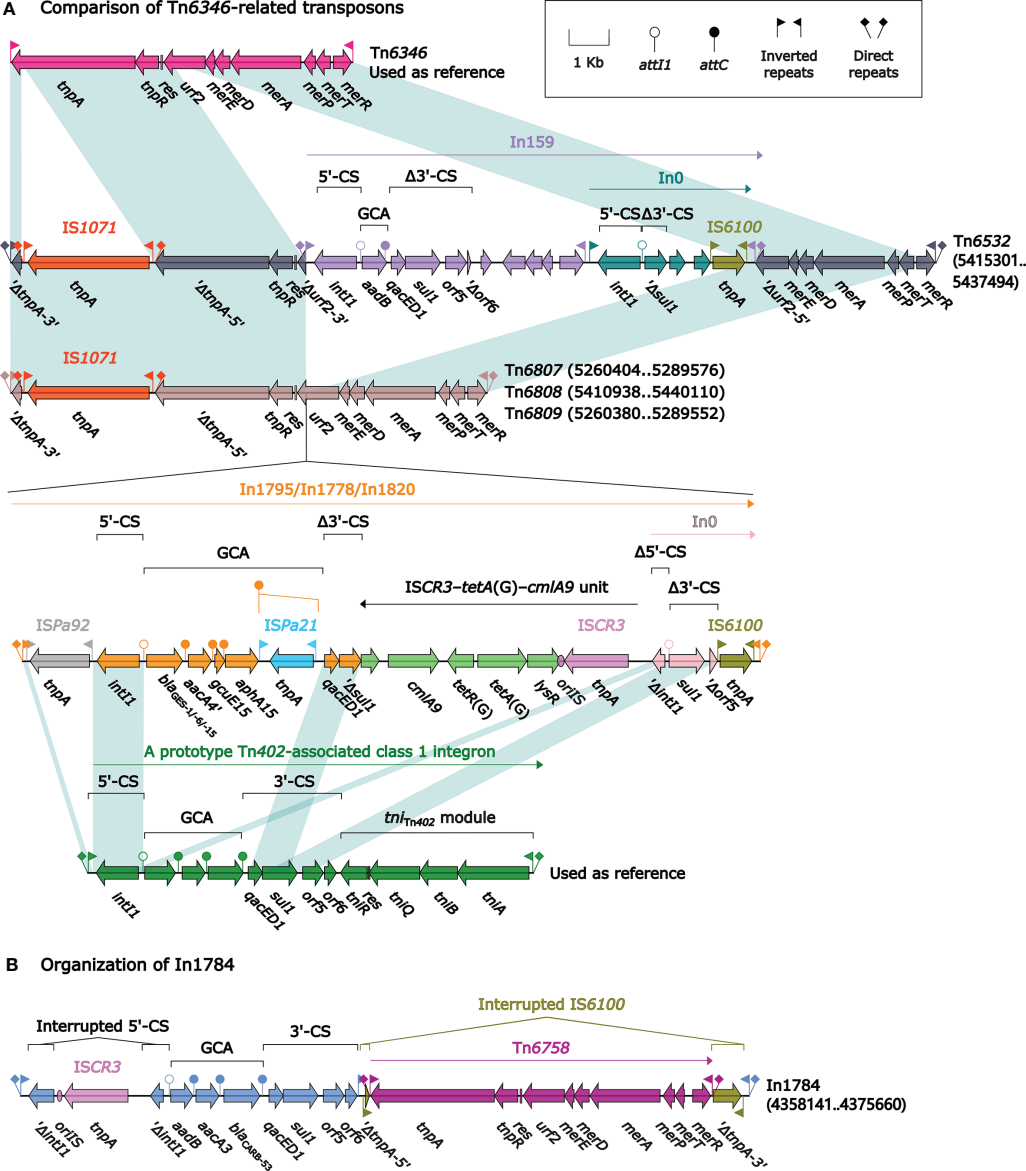
Comparison of Tn*6532*, Tn*6807*, Tn*6808* and Tn*6809*, and organization of In1784. Shown are five Tn*6346*-related transposons **(A)** and one integron In1784 **(B)**. Genes are denoted by arrows. Genes, MGEs, and other features are colored based on their functional classification. Shading denotes regions of homology (nucleotide identity ≥95%). Numbers in brackets indicate nucleotide positions within the chromosomes of strains DHS01, SE5352, SE5331, 31130, and 201330, respectively. The accession number of Tn*6346* (Ng et al., 2009) used as reference is EU696790.

Tn*6346*, a prototype Tn*3*-family unit transposon originally identified in *Achromobacter* spp. AO22 (Ng et al., 2009), was a hybrid of the core transposition module *tnpAR*–*res* from Tn*5051* and the *mer* region from Tn*501* (**Figure 3**). Tn*6532*, Tn*6807*, Tn*6808*, and Tn*6809* differed from Tn*6346* because of the interruption of *tnpA* due to the insertion of IS*1071* at the same position, and that of *urf2* due to the insertion of four different concise class 1 integrons In159, In1795, In1778, and In1820, respectively, at the same site; the four transposons Tn*6532*, Tn*6807*, Tn*6808*, and Tn*6809* were bracketed by the same 5-bp direct repeats (DRs; target site duplication signals for transposition).

In159 harbored the single-gene gene cassette array (GCA) *aadB*. In1795, In1778, and In1820 had the GCA *bla*_GES_–*aacA4′gcuE15*–*aphA15* but with differences in *bla*_GES_ subtypes: *bla*_GES-1_, *bla*_GES-6_, and *bla*_GES-15_, respectively. There were additional resistance loci besides GCAs integrated into these four integrons: an integron In0 into In159 and an IS*CR3–tetA*(G)*–cmlA9* unit connected with a different In0 into In1795/In1778/In1820. All these four integrons were bracketed by the same 5-bp DRs (**Figure 3**).

In1784 from Tn*6587* was a concise class 1 integron with the GCA *aadB*–*aacA3*–*bla*_CARB-53_. Its 5′-conserved segment (5′-CS: *intI1–attI1*) was interrupted by the insertion of IS*CR3*, leading to a 106-bp deletion of *intI1*. In addition, IS*6100* downstream of 3′-CS (*qacED1–sul1–orf5–orf6*) was interrupted by the insertion of Tn*6758* (Hu et al., 2015), which was a prototype Tn*3*-family unit transposon initially found in *Achromobacter xylosoxidans* X02736 and contained a core transposition module *tnpAR–res* plus a *mer* locus.

### Comparison of Four Related IMEs Tn*6852*, Tn*6853*, Tn*6854*, and Tn*6855*

**Figure 4:**
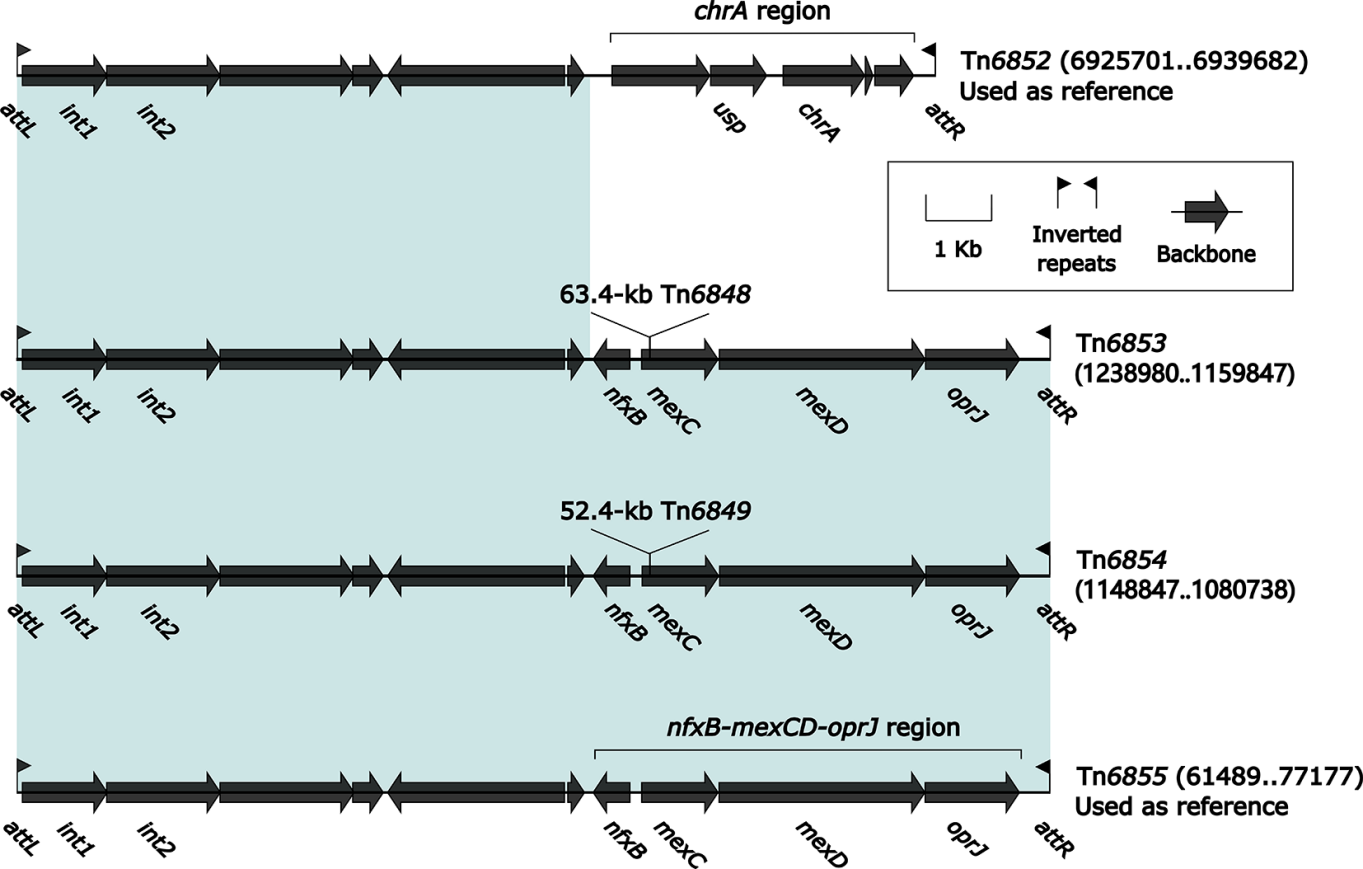
Organization of four related IMEs Tn*6852*, Tn*6853*, Tn*6854*, and Tn*6855*. Genes are denoted by arrows. Genes, MGEs, and other features are colored based on their functional classification. Shading denotes regions of homology (nucleotide identity ≥95%). Numbers in brackets indicate nucleotide positions within the chromosomes of strains PA38182, YT12746 and YTSEY8, and the plasmid pHNAH8I-1 of strain AHM7C8I, respectively. The accession number of Tn*6852* (Witney et al., 2014) used as reference is HG530068.

Tn6*852* (14.0 kb in length) was used as the prototype IME and initially found in *P. aeruginosa* PA38182 (Witney et al., 2014). Tn6*852* carried the backbone markers *attL/R*, *int*, and the *chrA* region (**Figure 4**). Conversely, Tn*6853*, Tn*6854*, and Tn*6855* (Lv et al., 2020) carried a multidrug efflux locus *nfxB*–*mexCD*–*oprJ* instead of the *chrA* region. Both the *chrA* region and the *nfxB*–*mexCD*–*oprJ* region were considered as the IME backbone components, since none of the associated MGEs were identified for them. Tn6*852* thereby had no accessory modules, but two Tn*3*-family unit transposons Tn*6848* and Tn*6849* as accessory modules were integrated at the same site within the backbone gene *mexC* of Tn*6853* and Tn*6854*, respectively. Tn*6852*, Tn*6853*, and Tn*6854* were integrated into the chromosomal gene *umuC* (DNA polymerase V subunit) in *P. aeruginosa*, while Tn*6855* into Tn*5393c* of plasmid pHNAH8I-1 in *K. pneumoniae*. Although plasmid-borne, Tn*6855* was included in this study because it was a prototype MGE carrying an intact *nfxB*–*mexCD*–*oprJ* locus that was often found in *Pseudomonas* species.

### Comparison of Five Related Unit Transposons Tn*1403*, Tn*6846*, Tn*6847*, Tn*6848*, and Tn*6849*

**Figure 5:**
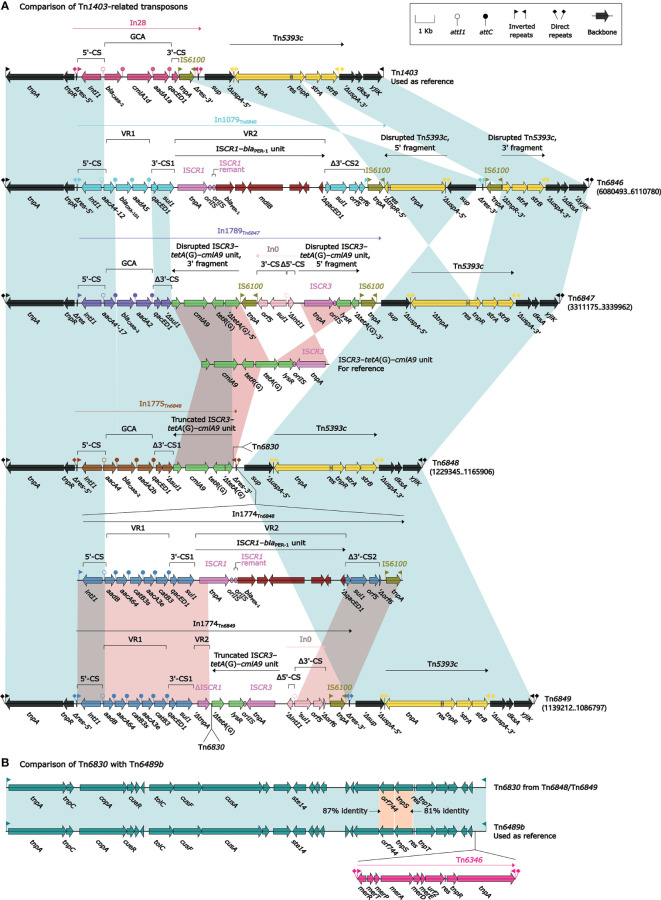
Organization of five related unit transposons Tn*1403*, Tn*6846*, Tn*6847*, Tn*6848*, and Tn*6849*. Shown are five Tn*1403*-related regions **(A)** and two Tn*6489b*-related transposons **(B)**. Genes are denoted by arrows. Genes, MGEs, and other features are colored based on their functional classification. Shading denotes regions of homology (nucleotide identity ≥95%). Numbers in brackets indicate nucleotide positions within the chromosomes of strains SE5357, YTSEY8, YT12746, and YTSEY8, respectively. The accession numbers of Tn*1403* (Stokes et al., 2007) and Tn*6489b* (Jiang et al., 2020) used as reference are AF313472 and MF344569, respectively.

The Tn*3*-family prototype unit transposon Tn*1403* was originally found in *P. aeruginosa* plasmid RPL11 (Levesque and Jacoby, 1988) and displayed a backbone structure IRL–*tnpAR*–*res*–*sup*–*uspA*–*dksA*–*yjiK*–IRR, with integration of two accessory modules In28 and Tn*5393c* into *res* and *uspA*, respectively (Stokes et al., 2007). Four Tn*1403* derivatives Tn*6846*, Tn*6847*, Tn*6848*, and Tn*6849* were identified in this study: the former two were chromosome-borne, Tn*6846* being integrated at a site upstream of the chromosomal gene *orf540* (thioesterase) while Tn*6847* being inserted into the chromosomal gene *orf1128* (glycerophosphodiester phosphodiesterase); the last two were located within the above-mentioned IMEs Tn*6853* and Tn*6854*, respectively (**Figure 5**). The primary modular difference in Tn*6846*, Tn*6847*, Tn*6848*, and Tn*6849* relative to Tn*1403* was the integration of different class 1 integrons In1079_Tn_*_6846_*, In1789_Tn_*_6847_*, In1775_Tn_*_6848_* (containing In1774_Tn_*_6848_*), and In1774_Tn_*_6849_*, instead of In28, into *res*. Another major modular difference was the occurrence of a 5.1-kb inversion in only Tn*6846*, and this inversion resulted from the insertion of IS*6100* into *tnpR*_Tn_*_5393c_* and led to the disruption of Tn*5393c* (*strAB* remained intact).

In1079_Tn_*_6846_* was a complex class 1 integron containing two resistance loci: GCA *aacA4-12*–*bla*_OXA-101_–*aacA5* (VR1: variable region 1), and IS*CR1*–*bla*_PER-1_ unit (VR2). The two concise class 1 integrons In1789_Tn_*_6847_* and In1775_Tn_*_6848_* harbored the distinct GCAs *aacA4′-17*–*bla*_CARB-2_–*aadA2* and *aacA4*–*bla*_CARB-2_–*aadA2b*, respectively and exhibited two additional major modular differences: i) a disrupted IS*CR3*–*tetA*(G)–*cmlA9* unit and another truncated version were inserted at the same site downstream of 3′-CS1, respectively; and ii) Tn*6830*–In1774_Tn_*_6848_* were integrated into the 3′-terminal region of In1775_Tn_*_6848_* but not In1789_Tn_*_6847_*. The two complex class 1 integrons In1774_Tn_*_6848_* and In1774_Tn_*_6849_* harbored the same GCA *aadB*–*aacA64*–*catB3s*–*aacA3e*–*catB3* (VR1), but contained two different VR2 regions: IS*CR1*–*bla*_PER-1_ unit and ΔIS*CR1*, respectively. Besides, a 31.9-kb fragment composed of Tn*6830*, a truncated IS*CR3*–*tetA*(G)–*cmlA9* unit, an empty integron In0 and IS*6100* was inserted into the 3′-terminal region of In1774_Tn_*_6849_*. Tn*6830* belonged to the Tn*4651* (Tsuda et al., 1989) subgroup of Tn*3*-family unit transposons, and it highly resembled Tn*6489b* (Jiang et al., 2020): Tn*6830* and Tn*6489b* shared a core transposition structure IRL–*tnpAC..tnpS*–*res*–*tnpT*–IRR, but Tn*6346* was integrated into Tn*6489b* but not into Tn*6830*.

### Comparison of Two Related IMEs Tn*6877* and Tn*6878* and Their Derivatives *dfrA12* Region and *bla*_VEB-3_ Region

**Figure 6:**
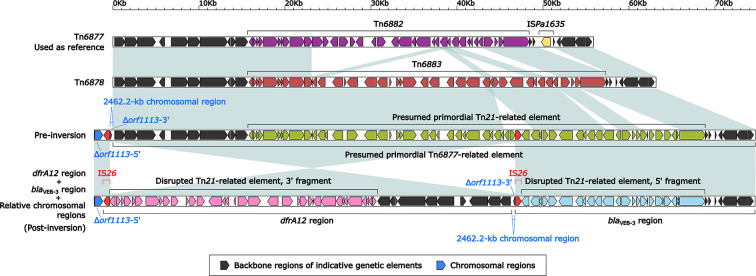
Organization of two related IMEs Tn*6877* and Tn*6878*, and their derivatives *dfrA12* region and *bla*_VEB-3_ region. Genes are denoted by arrows. Genes, MGEs, and other features are colored based on their functional classification. Shading denotes regions of homology (nucleotide identity ≥95%). The accession number of Tn*6877* (Espinosa-Camacho et al., 2017) used as reference is CP021775.

Tn6*877* (55.9 kb in length) was used as the prototype IME and initially found in *P. aeruginosa* Pa58 (Espinosa-Camacho et al., 2017). Tn*6877*, Tn*6878*, and a presumed primordial Tn6*877*-related element were all integrated at the same site between the two chromosomal genes *orf2892* (*α* subunit of ribonucleotide reductase) and *orf1248* (*ß* subunit), and they had essentially identical backbones, which was 21.6 kb in length and contained *attL/R* and two different *int* genes (**Figure 6**). Three Tn*21*-related accessory modules, namely Tn*6882*, Tn*6883*, and a presumed primordial Tn*21*-related element were inserted at the same site within the above three AGEs, respectively. In addition, Tn*6877* acquired the second accessory module IS*Pa1635*.

A huge DNA region composed of the *dfrA12* region plus a 2,462.2-kb chromosomal region underwent an inversion, which was likely mediated by two copies of IS*26*: one located within the primordial Tn6*877*-related element and another inserted into the chromosomal gene *orf1113* (chain length determinant protein) (**Figure 6**). This inversion split the primordial Tn6*877*-related element into two separate parts: the *dfrA12* region and the *bla*_VEB-3_ region; correspondingly, the primordial Tn*21*-related element in it was disrupted into the 5′-fragment and 3′-fragment.

**Figure 7:**
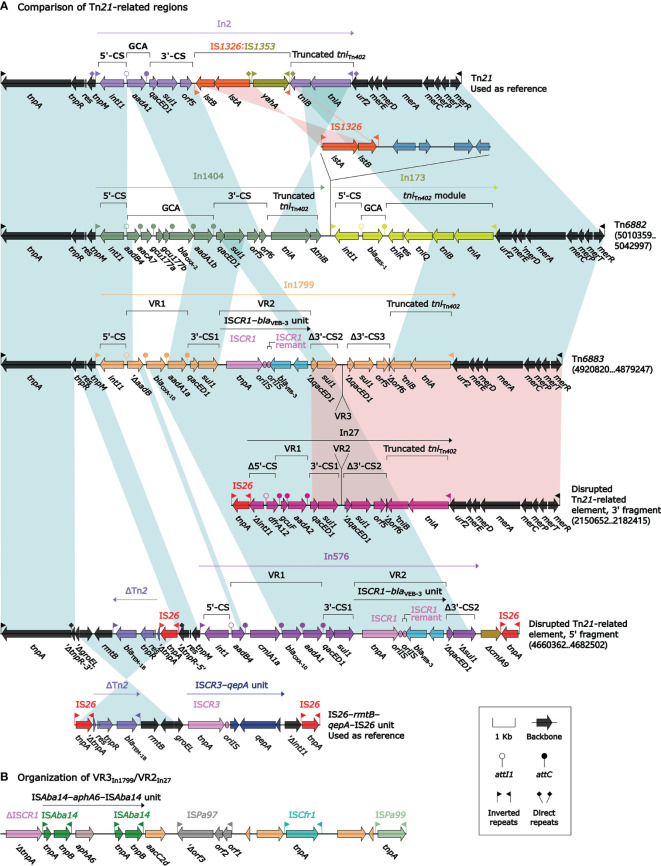
Comparison of Tn*6882*, Tn*6883*, and related regions. Shown are five T*n21*-related regions **(A)** and VR3_In1799_/VR2_In27_ **(B)**. Genes are denoted by arrows. Genes, MGEs, and other features are colored based on their functional classification. Shading denotes regions of homology (nucleotide identity ≥95%). Numbers in brackets indicate nucleotide positions within the chromosomes of strains Pa58, SE5429, and SE5458, respectively. The accession numbers of Tn*21* (Partridge et al., 2001) and IS*26*–*rmtB*–*qepA*–IS*26* unit (Yamane et al., 2007) used as reference are AF071413 and AB263754, respectively.

Tn*21* was a Tn*3*-family prototype unit transposon initially found in *Shigella flexneri* plasmid R100 and contained a core transposition module *tnpAR–res* plus a *mer* locus (Partridge et al., 2001). Tn*6882*, Tn*6883*, and the primordial Tn*21*-related element differed from Tn*21* by acquisition of In1404 + In173, In1799, and In576 + In27, respectively, instead of In2-3 (a class 2 integron with a single-gene GCA *dfrA1*); additionally, the *tnpR* gene of the primordial Tn*21*-related element was interrupted by the integration of a 4.4-kb truncated version of IS*26*–*rmtB*–*qepA*–IS*26* unit (**Figure 7**).

The concise class 1 integrons In1404 and In173 contained two different GCAs *aadB4*–*aacA7*–*gcu177a*–*gcu177b*–*bla*_OXA-2_–*aadA1b* and *bla*_GES-1_, respectively, as their sole resistance locus (**Figure 7**). The complex class 1 integrons In1799, In57, and In27 each carried multiple resistance loci. In1799 carried VR1 (GCA Δ*aadB*–Δ*cmlA1a*–*bla*_OXA-10_–*aadA1a*), VR2 (IS*CR1*–*bla*_VEB-3_ unit), and VR3 (a 17.5-kb region containing IS*Aba14*–*aphA6*–IS*Aba14* unit). In27 carried GCA *dfrA12*–*gcuF*–*aadA2* (VR1) and the 17.5-kb VR3_In1799_ region (VR2). In576 harbored GCA *aadB4*–*cmlA1a*–*bla*_OXA-10_–*aadA1* (VR1) and IS*CR1*–*bla*_VEB-3_ unit (VR2).

### Summary of Newly Identified or Designated MGEs

There were 20 newly identified MGEs, including four ICEs Tn*6584*, Tn*6585*, Tn*6586*, and Tn*6587*; three IMEs Tn*6853*, Tn*6854*, and Tn*6878*; five unit transposons Tn*6846*, Tn*6847*, Tn*6848*, Tn*6849*, and Tn*6883*; and eight integrons In1795, In1778, In1820, In1784, In1775, In1774, In1789, and In1799. Additional five MGEs (two IMEs Tn*6852* and Tn*6877*, one unit transposon Tn*6758*, and two ISs IS*Pa97* and IS*Pa99*) were newly designated (designated for the first time in this study but with previously determined sequences).

### Characterization of Two Novel Variants of Antibiotic Resistance Genes

This was the first report of a novel *bla*_CARB-2_ variant *bla*_CARB-53_ and a novel *catB3* variant *catB3s*. The deduced CARB-53 protein differs from CARB-2 by a single amino acid substitution Glu26Lys. *catB3s* differed from the *catB3* reference gene by one amino acid substitution Val210Ile.The *bla*_CARB-53_ or *catB3s* gene fragments were cloned into the cloning vector pUC57K and then transformed into *E. coli* TOP10 to obtain the electroporant TOP10/pUC57K-CARB or TOP10/pUC57K-catB.

As expected, TOP10/pUC57K-CARB was highly resistant to ampicillin, piperacillin, and carbenicillin but remained susceptible to cephalosporins and carbapenems (**Table 2**). TOP10/pUC57K-catB showed a very high level of resistance to chloramphenicol with a minimum inhibitory concentration (MIC) value ≥256, while the two negative control strains TOP10/pUC57K and TOP10 were susceptible to chloramphenicol with a MIC value of 8.

**Table 2:** Antimicrobial drug susceptibility profiles.

Antibiotics	MIC (μg/ml)/antimicrobial susceptibility		
	TOP10/pUC57K-CARB	TOP10/pUC57K	TOP10
Ampicillin	≥32/R	8/S	4/S
Piperacillin	≥128/R	≤4/S	≤4/S
Carbenicillin	>512/R	16/S	8/S
Cefazolin	≤4/S	≤4/S	≤4/S
Cefuroxime	8/S	8/S	8/S
Cefotetan	≤4/S	≤4/S	≤4/S
Ceftazidime	≤1/S	≤1/S	≤1/S
Ceftriaxone	≤1/S	≤1/S	≤1/S
Cefepime	≤1/S	≤1/S	≤1/S
Imipenem	≤1/S	≤1/S	≤1/S
Meropenem	≤0.25/S	≤0.25/S	≤0.25/S
Aztreonam	≤1/S	≤1/S	≤1/S

S, sensitive; R, resistant; I, intermediate.

Since the objective of this work was not to characterize these genes in terms of conferred phenotype, the cloning of the original genes was not done in parallel. Nevertheless, *bla*_CARB-53_ or *catB3s* had the drug resistance profile similar to its original variant *bla*_CARB-2_ (Korfhagen and Loper, 1975) or *catB3* (Bunny et al., 1995), respectively.

### ICE Transferability and Antimicrobial Susceptibility

A total of four ICEs Tn*6584*, Tn*6585*, Tn*6586*, and Tn*6587* were identified from the nine *P. aeruginosa* isolates, and all these four ICEs had the essential conjugal transfer genes. After repeated conjugation experiment attempts, only Tn*6587* was transferred from its rifampin-susceptible wild-type isolate into the rifampin-resistant *P. aeruginosa* PAO1, generating the transconjugant PAO1/Tn*6587*. PAO1/Tn*6587* was highly resistant to carbenicillin with a MIC value >512 owing to the presence of *bla*_CARB-53_, whereas PAO1 was susceptible to chloramphenicol with a MIC value of 32.

### Concluding Remarks

This work presented the complete sequences of 13 *P. aeruginosa* chromosomal AGEs, which could be divided into four groups: four Tn*6417*-related ICEs Tn*6584*, Tn*6585*, Tn*6586* and Tn*6587*; two Tn*6852*-related IMEs Tn*6853* and Tn*6854*; four Tn*1403*-related unit transposons Tn*6846*, Tn*6847*, Tn*6848* and Tn*6849*; and one Tn*6877*-related IME Tn*6878* and its two derivatives *dfrA12* region and *bla*_VEB-3_ region.

Ten of these 13 chromosomal AGEs carried 10 *β*-lactamase genes in total: four NSBL genes *bla*_CARB-2_, *bla*_CARB-53_, *bla*_OXA-101_, and *bla*_TEM-1B_ in Tn*6847*/Tn*6853* (containing Tn*6848*), Tn*6587*, Tn*6846*, and *bla*_VEB-3_ region, respectively; four ESBL genes *bla*_GES-1_, *bla*_PER-1_, and *bla*_VEB-3_/*bla*_OXA-10_ in Tn*6584*, Tn*6846*/Tn*6853* (containing Tn*6848*), and Tn*6878*/*bla*_VEB-3_ region, respectively; and two carbapenemase genes *bla*_GES-6_ and *bla*_GES-15_ in Tn*6585* and Tn*6586*, respectively. Notably, Tn*6853* (containing Tn*6848*), Tn*6878*, *bla*_VEB-3_ region, and Tn*6846* each harbored two different *β*-lactamase genes.

Of the 10 *β*-lactamase genes in the 10 chromosomal AGEs sequenced in this study, nine (except for *bla*_TEM-1B_ in a truncated IS*26*–*rmtB*–*qepA*–IS*26* unit) were associated with class 1 integrons. The first seven *β*-lactamase genes *bla*_CARB-2/-53_, *bla*_OXA-10/-101_, and *bla*_GES-1/-6/-15_ were located in the GCAs of In1789/In1775/In1784, In1799/In1079, and In1795/In1778/In1820, respectively; conversely, the remaining two *bla*_PER-1_ and *bla*_VEB-3_ were located in the VRs of complex integrons In1079 and In576/In1799, respectively. These integrons, which captured various *β*-lactamase genes, were further integrated not only relevant into ICEs and IMEs with intercellular mobility but also into relevant unit transposons with intracellular mobility.

Besides the above 10 *β*-lactamase genes, there were additional 35 resistance genes identified in various subregions (including integrons, unit transposons, and putative resistance units) of these 13 chromosomal AGEs; thereby, most of these chromosomal AGEs had mosaic modular structures and encoded multiple drug resistance (**Table S2**).

Data presented here denoted that complex events of transposition and homologous recombination promoted the assembly and further integration of these chromosomal AGEs, carrying a large amount of resistance genes, into *P. aeruginosa* chromosomes.

## Data Availability Statement

The datasets generated for this study can be found in the complete nucleotide sequences of SE5352, SE5331, 31130, 201330, YT12726, YTSEY8, SE5429, SE5458 and SE5357 isolates, which were submitted to GenBank under accession numbers CP054843, CP046402, CP060392, CP054623, CP045552, CP054581, CP054845, CP046406 and CP054844, respectively.

## Ethics Statement

This study uses the clinical bacterial isolates obtained from the Chinese public hospitals as listed in **Table S1**. The local legislation did not require the study to be reviewed or approved by an ethics committee because the bacterial isolates involved in this study were part of the routine hospital laboratory procedures. The research involving biohazards and all related procedures were approved by the Biosafety Committee of the Beijing Institute of Microbiology and Epidemiology.

## Author Contributions

DZ and MZ conceived the study and designed experimental procedures. TY, YJ, and FC performed the experiments. TY, LH, and XL analyzed the data. HY, JL, and ZY contributed to reagents and materials. TY and HY wrote the original draft. DZ and MZ reviewed the manuscript. All authors contributed to the article and approved the submitted version.

## Funding

This work was supported by the National Key R&D Program of China under grant number 2018YFC1200100 and the Fundamental Research Funds for the Central Universities of Central South University under grant number 502211911 (2019zzts796).

## Conflict of Interest

The authors declare that the research was conducted in the absence of any commercial or financial relationships that could be construed as a potential conflict of interest.
