# Supplementary material for: Novel Chromosome-Borne Accessory Genetic Elements Carrying Multiple Antibiotic Resistance Genes in Pseudomonas aeruginosa
Source: Front Cell Infect Microbiol. 2021 Mar 18;11:638087. doi: 10.3389/fcimb.2021.638087 (PMC8012812; doi:10.3389/fcimb.2021.638087)
Supplement: Supplementary file 2 [file Table_2.docx]

**Table S2. Resistance genes in the genetic elements characterized in this work**

| **Group** | **Genetic element** | **Resistance marker** | **Resistance phenotype** | **Nucleotide position** | **Subregion located** |
| --- | --- | --- | --- | --- | --- |
| Tn*6417*-related ICEs | Tn*6417* | *aadB* | Aminoglycoside resistance | 5423757..5424290 | Tn*6532* |
|  |  | *qacED1* | Quaternary ammonium  compound resistance | 5424447..5424794 |  |
|  |  | *sul1* | Sulphonamide resistance | 5424788..5425627 |  |
|  |  | *mer* locus | Mercuric resistance | 5433944..5437461 |  |
|  | Tn*6584* | *bla*_GES-1_ | β-lactam resistance | 5270485..5271348 | Tn*6807* |
|  |  | *aacA4'* | Aminoglycoside resistance | 5271487..5272041 |  |
|  |  | *aphA15* | Aminoglycoside resistance | 5272374..5273168 |  |
|  |  | *qacED1* | Quaternary ammonium  compound resistance | 5274758..5275105 |  |
|  |  | *sul1* | Sulphonamide resistance | 5283031..5283882 |  |
|  |  | *cmlA9* | Phenicol resistance | 5276287..5277501 |  |
|  |  | *tetA*(G) | Tetracycline resistance | 5278438..5279613 |  |
|  |  | *mer* locus | Mercuric resistance | 5286026..5289543 |  |
|  | Tn*6585* | *bla*_GES-6_ | β-lactam resistance | 5421019..5421882 | Tn*6808* |
|  |  | *aacA4'* | Aminoglycoside resistance | 5422021..5422575 |  |
|  |  | *aphA15* | Aminoglycoside resistance | 5422908..5423702 |  |
|  |  | *qacED1* | Quaternary ammonium  compound resistance | 5425292..5425639 |  |
|  |  | *sul1* | Sulphonamide resistance | 5433565..5434416 |  |
|  |  | *cmlA9* | Phenicol resistance | 5426821..5428035 |  |
|  |  | *tetA*(G) | Tetracycline resistance | 5428972..5430147 |  |
|  |  | *mer* locus | Mercuric resistance | 5436560..5440077 |  |
|  | Tn*6586* | *bla*_GES-15_ | β-lactam resistance | 5270461..5271324 | Tn*6809* |
|  |  | *aacA4'* | Aminoglycoside resistance | 5271463..5272017 |  |
|  |  | *aphA15* | Aminoglycoside resistance | 5272350..5273144 |  |
|  |  | *qacED1* | Quaternary ammonium  compound resistance | 5274734..5275081 |  |
|  |  | *sul1* | Sulphonamide resistance | 5283007..5283858 |  |
|  |  | *cmlA9* | Phenicol resistance | 5276263..5277477 |  |
|  |  | *tetA*(G) | Tetracycline resistance | 5278414..5279589 |  |
|  |  | *mer* locus | Mercuric resistance | 4081494..4081860 |  |
|  | Tn*6587* | *bla*_CARB-53_ | β-lactam resistance | 4363031..4363897 | In1784 |
|  |  | *aadB* | Aminoglycoside resistance | 4361752..4362285 |  |
|  |  | *aacA3* | Aminoglycoside resistance | 4362359..4362913 |  |
|  |  | *qacED1* | Quaternary ammonium  compound resistance | 4364114..4364461 |  |
|  |  | *sul1* | Sulphonamide resistance | 4364455..4365294 |  |
|  |  | *mer* locus | Mercuric resistance | 4371203..4374723 |  |
| Tn*6852*-related IMEs | Tn*6852* | *chrA* | Chromate resistance | 6937366..6938607 | IME backbone |
|  | Tn*6853* | *aacA4* | Aminoglycoside resistance | 1223618..1224292 | Tn*6848* |
|  |  | *bla*_CARB-2_ | β-lactam resistance | 1222622..1223536 |  |
|  |  | *aadA2b* | Aminoglycoside resistance | 1221713..1222492 |  |
|  |  | *qacED1* | Quaternary ammonium  compound resistance | 1221202..1221549  1187313..1187660 |  |
|  |  | *sul1* | Sulphonamide resistance | 1186480..1187319  1177500..1178339 |  |
|  |  | *cmlA9* | Phenicol resistance | 1218806..1220020 |  |
|  |  | *aadB* | Aminoglycoside resistance | 1190575..1191108 |  |
|  |  | *aacA64* | Aminoglycoside resistance | 1189944..1190495 |  |
|  |  | *catB3s* | Phenicol resistance | 1189160..1189792 |  |
|  |  | *catB3* | Phenicol resistance | 1187817..1188449 |  |
|  |  | *aacA3e* | Aminoglycoside resistance | 1188532..1189086 |  |
|  |  | *bla*_PER-1_ | β-lactam resistance | 1183285..1184211 |  |
|  |  | *cop* locus | Copper resistance | 1211283..1213739 |  |
|  |  | *strA* | Aminoglycoside resistance | 1169139..1169942 |  |
|  |  | *strB* | Aminoglycoside resistance | 1168303..1169139 |  |
|  | Tn*6854* | *aadB* | Aminoglycoside resistance | 1133583..1134116 | Tn*6849* |
|  |  | *aacA64* | Quaternary ammonium  compound resistance | 1132952..1133503 |  |
|  |  | *catB3s* | Phenicol resistance | 1132168..1132800 |  |
|  |  | *aacA3e* | Aminoglycoside resistance | 1131540..1132094 |  |
|  |  | *catB3* | Phenicol resistance | 1130825..1131457 |  |
|  |  | *qacED1* | Quaternary ammonium  compound resistance | 1130321..1130668 |  |
|  |  | *sul1* | Sulphonamide resistance | 1129488..1130327 |  |
|  |  | *cop* locus | Copper resistance | 1122181..1124636 |  |
|  |  | *strA* | Aminoglycoside resistance | 1090030..1090832 |  |
|  |  | *strB* | Aminoglycoside resistance | 1089194..1090030 |  |
|  | Tn*6855* | None | | | |
| Tn*1403*-related  unit transposons | Tn*1403* | *bla*_CARB-2_ | β-lactam resistance | 5158..6024 | In28 |
|  |  | *cmlA1d* | Phenicol resistance | 6339..7598 |  |
|  |  | *aadA1a* | Aminoglycoside resistance | 7691..8482 |  |
|  |  | *strA* | Aminoglycoside resistance | 15594..16397 | Tn*5393c* |
|  |  | *strB* | Aminoglycoside resistance | 16397..17233 |  |
|  | Tn*6846* | *aacA4-12* | Aminoglycoside resistance | 6085591..6086145 | In1079 |
|  |  | *bla*_OXA-101_ | β-lactam resistance | 6086226..6087026 |  |
|  |  | *aadA5* | Aminoglycoside resistance | 6087088..6087876 |  |
|  |  | *qacED1* | Quaternary ammonium  compound resistance | 6088044..6088391 |  |
|  |  | *sul1* | Sulphonamide resistance | 6088385..6089224  6097365..6098204 |  |
|  |  | *bla*_PER-1_ | β-lactam resistance | 6091493..6092419 |  |
|  |  | *strA* | Aminoglycoside resistance | 6106786..6107589 | Tn*5393c* |
|  |  | *strB* | Aminoglycoside resistance | 6107589..6108425 |  |
|  | Tn*6847* | *aacA4'-17* | Aminoglycoside resistance | 3316287..3316841 | In1789 |
|  |  | *bla*_CARB-2_ | β-lactam resistance | 3316971..3317837 |  |
|  |  | *aadA2* | Aminoglycoside resistance | 3317967..3318746 |  |
|  |  | *qacED1* | Quaternary ammonium  compound resistance | 3318910..3319257 |  |
|  |  | *sul1* | Sulphonamide resistance | 3324892..3325743 |  |
|  |  | *cmlA9* | Phenicol resistance | 3320439..3321653 |  |
|  |  | *strA* | Aminoglycoside resistance | 3335926..3336729 | Tn*5393c* |
|  |  | *strB* | Aminoglycoside resistance | 3336729..3337565 |  |
|  | Tn*6848* | *aacA4* | Aminoglycoside resistance | 1223618..1224292 | In1775 |
|  |  | *bla*_CARB-2_ | β-lactam resistance | 1222622..1223536 |  |
|  |  | *aadA2b* | Aminoglycoside resistance | 1221713..1222492 |  |
|  |  | *qacED1* | Quaternary ammonium  compound resistance | 1221202..1221549  1187313..1187660 |  |
|  |  | *sul1* | Sulphonamide resistance | 1186480..1187319  1177500..1178339 |  |
|  |  | *cmlA9* | Phenicol resistance | 1218806..1220020 |  |
|  |  | *aadB* | Aminoglycoside resistance | 1190575..1191108 |  |
|  |  | *aacA64* | Aminoglycoside resistance | 1189944..1190495 |  |
|  |  | *catB3s* | Phenicol resistance | 1189160..1189792 |  |
|  |  | *catB3* | Phenicol resistance | 1187817..1188449 |  |
|  |  | *aacA3e* | Aminoglycoside resistance | 1188532..1189086 |  |
|  |  | *bla*_PER-1_ | β-lactam resistance | 1183285..1184211 |  |
|  |  | *cop* locus | Copper resistance | 1211283..1213739 |  |
|  |  | *strA* | Aminoglycoside resistance | 1169139..1169942 | Tn*5393c* |
|  |  | *strB* | Aminoglycoside resistance | 1168303..1169139 |  |
|  | Tn*6849* | *aadB* | Aminoglycoside resistance | 1133583..1134116 | In1774 |
|  |  | *aacA64* | Aminoglycoside resistance | 1132952..1133503 |  |
|  |  | *catB3s* | Phenicol resistance | 1132168..1132800 |  |
|  |  | *aacA3e* | Aminoglycoside resistance | 1131540..1132094 |  |
|  |  | *catB3* | Phenicol resistance | 1130825..1131457 |  |
|  |  | *qacED1* | Quaternary ammonium  compound resistance | 1130321..1130668 |  |
|  |  | *sul1* | Sulphonamide resistance | 1129488..1130327 |  |
|  |  | *cop* locus | Copper resistance | 1122181..1124636 |  |
|  |  | *strA* | Aminoglycoside resistance | 1090030..1090832 | Tn*5393c* |
|  |  | *strB* | Aminoglycoside resistance | 1089194..1090030 |  |
| Tn*6877*-related IMEs | Tn*6877* | *bla*_GES-1_ | β-lactam resistance | 5019838..5020701 | Tn*6882* |
|  |  | *aadB4* | Aminoglycoside resistance | 5037064..5037597 |  |
|  |  | *aacA7* | Aminoglycoside resistance | 5036547..5037005 |  |
|  |  | *bla*_OXA-2_ | β-lactam resistance | 5034770..5035597 |  |
|  |  | *aadA1b* | Aminoglycoside resistance | 5033942..5034733 |  |
|  |  | *qacED1* | Quaternary ammonium  compound resistance | 5033431..5033778 |  |
|  |  | *sul1* | Sulphonamide resistance | 5032598..5033437 |  |
|  |  | *mer* locus | Mercuric resistance | 5010392..5014354 |  |
|  | Tn*6878* | *bla*_OXA-10_ | β-lactam resistance | 4913786..4914586 | Tn*6883* |
|  |  | *aadA1a* | Aminoglycoside resistance | 4912886..4913677 |  |
|  |  | *qacED1* | Quaternary ammonium  compound resistance | 4912375..4912722 |  |
|  |  | *sul1* | Sulphonamide resistance | 4911542..4912381  4906457..4907296  4887292..4888131 |  |
|  |  | *bla*_VEB-3_ | β-lactam resistance | 4908434..4909333 |  |
|  |  | *aphA6* | Aminoglycoside resistance | 4902302..4903081 |  |
|  |  | *aacC2d* | Aminoglycoside resistance | 4899245..4900105 |  |
|  |  | *mer* locus | Mercuric resistance | 4879280..4883242 |  |
|  | *dfrA12* region | *dfrA12* | Trimethoprim resistance | 2152164..2152661 | Disrupted Tn*21*-related element, 3' fragment |
|  |  | *aadA2* | Aminoglycoside resistance | 2153081..2153860 |  |
|  |  | *qacED1* | Quaternary ammonium  compound resistance | 2154024..2154371 |  |
|  |  | *sul1* | Sulphonamide resistance | 2154365..2155204  2173531..2174370 |  |
|  |  | *aphA6* | Aminoglycoside resistance | 2158581..2159360 |  |
|  |  | *aacC2d* | Aminoglycoside resistance | 2161557..2162417 |  |
|  |  | *mer* locus | Mercuric resistance | 2178420..2182382 |  |
|  | *bla*_VEB-3_ region | *bla*_VEB-3_ | β-lactam resistance | 4664330..4665229 | Disrupted Tn*21*-related element, 5' fragment |
|  |  | *sul1* | Sulphonamide resistance | 4667438..4668277 |  |
|  |  | *qacED1* | Quaternary ammonium  compound resistance | 4668271..4668618 |  |
|  |  | *aadA1* | Aminoglycoside resistance | 4668782..4669582 |  |
|  |  | *bla*_OXA-10_ | β-lactam resistance | 4669682..4670482 |  |
|  |  | *cmlA1a* | Phenicol resistance | 4670586..4671845 |  |
|  |  | *aadB4* | Aminoglycoside resistance | 4672100..4672633 |  |
|  |  | *bla*_TEM-1_ | β-lactam resistance | 4676731..4677591 |  |
|  |  | *rmtB* | Aminoglycoside resistance | 4677761..4678516 |  |
